# Exploring the psychosocial implications of general population screening for paediatric type 1 diabetes in Europe: Protocol for a mixed‐methods study

**DOI:** 10.1111/dme.70297

**Published:** 2026-04-03

**Authors:** O. Boiko, K. Barnard‐Kelly, J. Vercauteren, S. M. Greenfield, R. P. Dias, L. M. Quinn, I. Litchfield, T. Papanikolaou, J. L. Dunne, G. Perego, F. Gatti, F. Milano, B. M. Milazzo, V. Di Mattei, K. Lange, C. Mathieu, L. Overbergh, A.‐G. Ziegler, C. Hurtado Del Pozo, M. G. Scarale, A. Mahieu, A. J. Omar Alsaleh, E. Bosi, P. Narendran, EDENT1FI Consortium, C. Mathieu, P. Gillard, K. Casteels, B. van der Schueren, L. Overbergh, J. Vercauteren, A. G. Ziegler, P. Achenbach, F. Haupt, C. Winkler, S. Hummel, F. Reschke, O. Kordonouri, T. von dem Berge, K. Lange, T. Pieber, J. Mader, S. Moitzi, S. Del Prato, B. Torbeyns, Z. Sumnik, O. Cinek, V. Neuman, B. Berka, F. Pociot, B. Petersen, J. Antvorskov, J. H. Klaebel, R. Lahesmaa, H. Hyöty, J. E. Laiho, F. Reschke, O. Kordonouri, T. von dem Berge, E. Bonifacio, R. Berner, A. Hommel, A. Loff, G. Gemulla, N. Zubizarreta, E. Bosi, L. Piemonti, V. Lampasona, F. Dotta, G. Sebastiani, A. Szypowska, K. Karczewski, P. Jarosz‐Chobot, J. F. Raposo, R. Ribeiro, R. Coelho, M. Peakman, E. Niemoeller, M. Baccara‐Dinet, J. Van Rampelbergh, R. Bergholdt, K. Fogh, O. Cohen, M. I. Buompensiere, C. Hurtado del Pozo, E. Latres, G. Agiostratidou, A. Koralova, J. Jackson, D. Darrock, H. Tewson, S. Nagallas, J. Hedrick, K. Collins, T. Tree, C. Dayan, K. Hood, J. Townson, R. Playle, R. Besser, P. Narendran, I. Litchfield, S. Greenfield, R. Dias

**Affiliations:** ^1^ Department of Applied Health Sciences College of Medicine and Health, University of Birmingham Birmingham UK; ^2^ Southern Health NHS Foundation Trust Southampton UK; ^3^ Department of Chronic Diseases and Metabolism KU Leuven Leuven Belgium; ^4^ Institute of Immunology and Immunotherapy, College of Medicine and Health University of Birmingham Birmingham UK; ^5^ Sanofi US Bridgewater New Jersey USA; ^6^ School of Psychology Vita‐Salute San Raffaele University Milan Italy; ^7^ Department of Psychology University of Milan‐Bicocca Milan Italy; ^8^ Department of Medical Psychology Hannover Medical School Hannover Germany; ^9^ Laboratory of Clinical and Experimental Endocrinology Katholieke Universiteit Leuven (KULEUVEN) Leuven Belgium; ^10^ Institute of Diabetes Research, Helmholtz Munich, and Forschergruppe Diabetes, Klinikum Rechts der Isar Technical University Munich Munich Germany; ^11^ Research Department Breakthrough T1D New York City New York USA; ^12^ University Centre for Statistics in the Biomedical Sciences (CUSSB) Vita‐Salute San Raffaele University Milan Italy; ^13^ Sanofi France Gentilly France; ^14^ Sanofi Italy Milan Italy; ^15^ General Medicine, Diabetes and Endocrinology Department, Diabetes Research Institute at Ospedale San Raffaele Università Vita‐Salute San Raffaele Milan Italy

**Keywords:** children, diabetes mellitus type 1, psychosocial, qualitative interviews, screening, surveys and questionnaires

## Abstract

**Aims:**

Research and policy initiatives for type 1 diabetes (T1D) across European countries demonstrate an emergent, critical shift towards universal paediatric screening. National adaptations of screening, however, remain under‐researched. The current study, as part of the EU‐funded EDENT1FI programme, aimed to examine the psychosocial impact and acceptability of screening according to local contexts and regions.

**Methods:**

The EDENT1FI screening research programme includes 27 partners involved in implementing screening across eight European countries—four countries already implementing national or local research screening programmes and four countries that are new adopters. The study consists of four sub‐studies with assessments performed at five timepoints, from screening baseline to insulin‐requiring T1D: (1) we examine the screening‐specific psychological impact on families using a novel questionnaire for parents of screened children; (2) we further measure the psychological wellbeing of parents by administering validated questionnaires; (3) we explore parental acceptability through semi‐structured interviews and (4) we explore professional stakeholder acceptability via semi‐structured interviews. Data analysis will integrate questionnaire responses and thematic analysis for the interviews.

**Results:**

Findings should provide a deeper understanding of the psychosocial aspects of universal paediatric screening measured longitudinally. The experience of implementation context across countries will be harmonised with the attitudes towards and practices of screening to inform best practice for scaling up universal screening.

**Conclusions:**

The study will provide essential insights into how best a general population screening program for T1D can be integrated into existing European health systems.

## 1 BACKGROUND

Type 1 diabetes (T1D) is a chronic autoimmune inflammatory condition resulting in loss of insulin secreting beta cells and the need subsequently for lifelong insulin therapy.^1^ Across Europe, 300,000 children and adolescents live with T1D,^2^ with associated annual treatment costs of 30 billion Euros to local healthcare systems. Detecting T1D early, and prior to the need for insulin therapy, has significant advantages including better long‐term glucose control,^3^ time to prepare for insulin therapy,^4^, ^5^ and significantly reduced risk of being diagnosed in a diabetic emergency.^6^, ^7^ T1D can now be detected in the presymptomatic stages through the measurement of islet‐specific autoantibodies (Aab) and oral glucose tolerance testing^8^ thus raising the possibility of general population screening for this condition. Furthermore, guidelines are now available for clinically managing individuals identified at these early stages through periodic medical monitoring, education about symptoms of diabetes and psychosocial support.^8^

Screening studies (Table 1), clinical trials and recent national legislations^9^ endorsed for presymptomatic T1D across European countries suggest an emergent, critical shift in the expert and public discourse towards increased uptake of universal paediatric screening. This shift is underpinned by an increasing prevalence of T1D in children,^10^ introduction of less invasive testing, development of staging for presymptomatic T1D^11^ and the progressive licensing of a new immunoprevention therapy, teplizumab, in the US.^12^

**TABLE 1:** Recent and ongoing European paediatric screening initiatives.

Program title	Study characteristics	Primary location	Web‐link
DiaUnion 1.0, (2.0 underway)	Two‐centre, 4000 children screened for biomarkers for T1D, coeliac disease, autoimmune thyroid disease	Capital Region (Denmark) and Region Skåne (Sweden)	https://diaunion.org/en/research/#DiaUnion_20
ELSA	Multi‐centre, 20,000+ children screened for T1D	West Midlands (United Kingdom)	https://elsadiabetes.nhs.uk
Fr1da	Multi‐centre, 200,000+ children screened for T1D	Bavaria (Germany)	https://www.typ1diabetes‐frueherkennung.de
D1Ce	Multi‐centre, 5363 children screened for T1D and coeliac disease	Four regions: Lombardy, Marche, Campania, Sardinia (Italy)	https://www.iss.it/‐/il‐progetto‐d1ce

**FIGURE 1:**
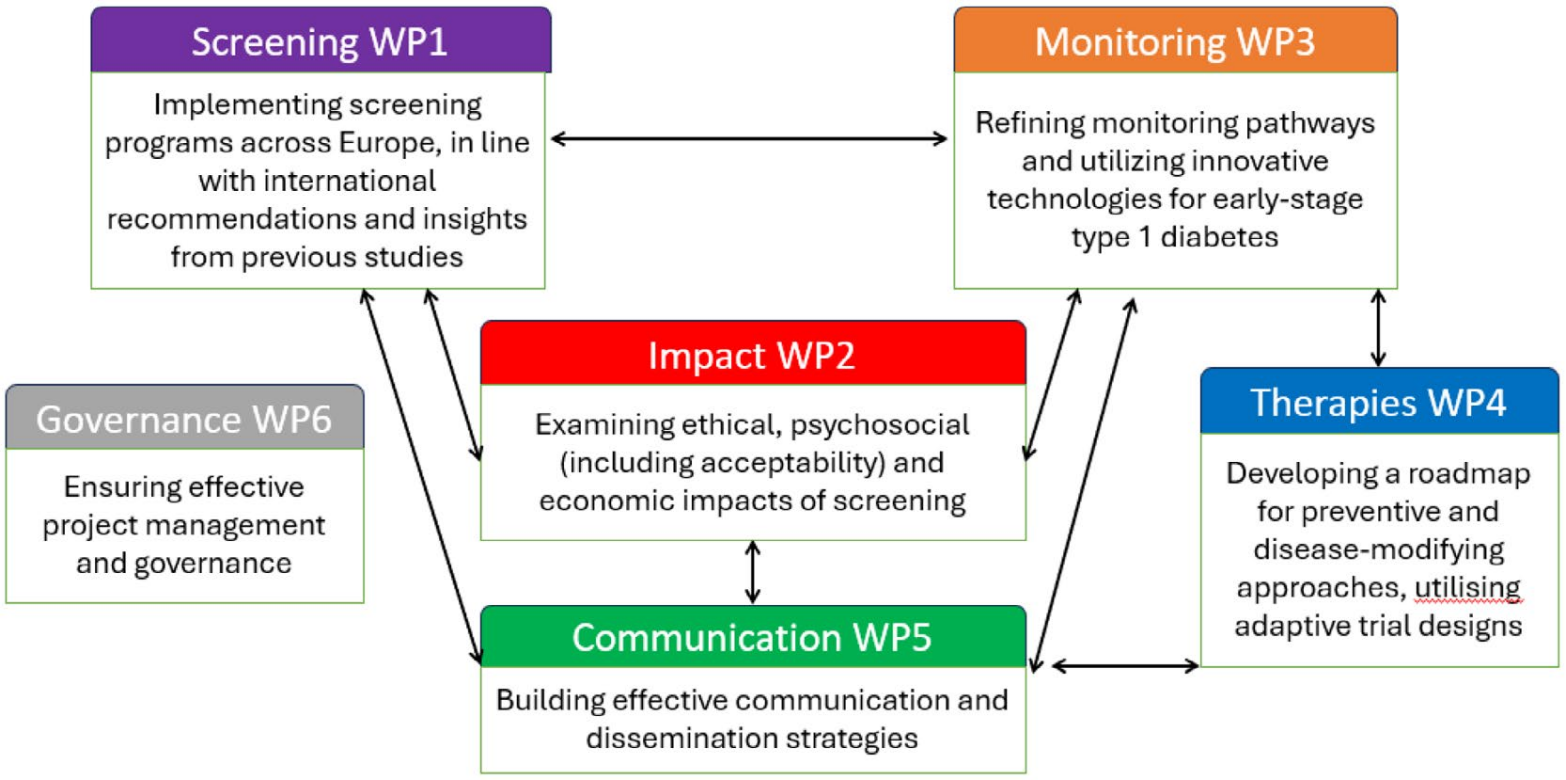
Flow chart of Work Packages for the EDENT1FI programme. The EDENT1FI programme comprises six work packages (WPs), which (1) establish screening programs across Europe, aligning with international recommendations and leveraging insights from previous studies (‘Screening WP’); (2) examine ethical, psychosocial (including acceptability) and economic impacts of screening (‘Impact WP’); (3) focus on refining monitoring pathways and utilising innovative technologies for early‐stage T1D (‘Monitoring WP’); (4) develop a roadmap for preventive and disease‐modifying approaches, utilising adaptive trial designs (‘Therapies WP’); (5) emphasize effective communication and dissemination strategies (‘Communication WP’) and (6) ensure effective project management and governance (‘Governance WP’).

The **E**uropean Action for the **D**iagnosis of **E**arly Non‐clinical **T1**D **F**or Disease **I**nterception (EDENT1FI) is composed of 27 partners across 13 countries, including academic, industrial and patient organisations. Tailored to individual country health systems, EDENT1FI aims to establish harmonised screening programmes, assess the impact of screening on diverse populations, refine monitoring protocols, develop innovative therapeutic strategies and disseminate findings to stakeholders. The project comprises six work packages (WPs) (Figure 1).

The protocol for the screening programme (Screening WP1) has been recently published.^13^ The protocol outlined in this current manuscript describes the study aiming to explore the psychosocial impact of general population screening (Impact WP2). This study will be conducted across eight European countries, some of which have established general population screening research programmes (Germany, henceforth called ‘Level 3’ country); some have nascent research programmes (United Kingdom, Denmark, Sweden, henceforth called level ‘2 countries’) and others will be starting their screening programmes as part of the EDENT1FI study (‘Level 1’ countries, Czechia, Italy, Poland, Portugal).

The balance of benefits and risks of population screening has drawn recent attention. Clinical outcomes were most often evidenced with drastic (5–10 times) reduced risk of diabetic ketoacidosis (DKA) at clinical manifestation of the disease for screened children.^7^ From the parents' perspective, some psychological trade‐offs were registered and some of these were related to anxiety and depressive symptoms. In ASK, DiPiS, GPPAD and Fr1da, identifying a child with islet‐specific Aab was associated with higher anxiety compared to having an Aab‐negative child, particularly in mothers. Following screening results notification, the German Fr1da study reported significantly increased median PHQ‐9 score in mothers of children with presymptomatic T1D compared to negative controls.^6^ Similarly, in the US‐based ASK study, anxiety level (SAI) in parents of autoantibody‐positive children rose following screening: 74% experienced SAI above clinical threshold at first follow‐up visit.^14^ Furthermore, anxiety arising from waiting for the onset of symptomatic T1D requiring insulin may lead to overly protective behaviours in parents.^4^, ^6^ In a newborn genetic testing study, increased maternal worry was found if the mother herself had type 1 diabetes.^15^

Conversely, psychosocial benefits relate to families of being aware of future diabetes risk. The TEDDY study, an international surveillance T1D program for genetically predisposed children, established that parents (*n* = 54) who knew of their child's positive Aab status demonstrated a decrease in stress in the first year post‐diagnosis and improved quality of life compared to those (*n* = 54) diagnosed in the community.^16^ This suggests a trend towards waning anxiety with time and possibly related to monitoring and support. However, in ASK, at visit 2–6 months after results notification, despite a significant decrease, 69% of parents reported clinically relevant anxiety.^14^

In terms of uptake, in TEDDY, enrolment was high for first degree relatives (65%) but lower from the general population (39%).^17^ Parents' satisfaction was high.^18^, ^19^, ^20^ Parents perceived benefits to outweigh harms^5^, ^21^ and twelve years after screening, parents perceived no adverse effects.^18^ With regards to (dis)continuation, adherence with the monitoring protocol in TEDDY was high at 62%, with large differences across countries,^22^ while in the longitudinal ABIS study, participation was lower at 43.8% at 5–6 years.^23^

Therefore, there appears to be mixed results around population screening. Since the transient character of parental anxiety was established as ‘fading’ with time emotional response,^16^ new investigations tailored to the emotional states resulting from presymptomatic T1D have been called for.^8^ The psychological status needs to be monitored with tailored measures and longitudinally, particularly for families identified to be at risk from the general population. Importantly, these findings need to be distinguished from pre‐existing anxieties and stress from other life events. There is clear need for improved understanding of professional stakeholders' acceptability. A recent guidance suggested careful implementation: by nominating a champion for the program, building a team to implement screening, motivating other providers to participate, integrating screening into existing workflows and streamlining logistics such as ordering and coding for autoantibody panels^24^; however, before improving feasibility, a better understanding of the professionals' views and readiness is warranted. Further insight into the issues associated with acceptability of national population screening programs in countries where implementation is on the rise is required. Finally, European countries' variation in screening programs should be accounted for: what screening ‘is’ in any one country or region reflects pragmatic realities of implementation as well as public attitudes and acceptability.

## 2 AIMS

Our overarching aim is to explore the psychosocial impact and acceptability of general population screening for T1D in a regional and culturally sensitive manner.

Our specific aims are:

1. Evaluate the psychological impact of paediatric T1D screening through established validated self‐reported questionnaires and through the development of a novel questionnaire.
2. Examine the psychosocial impact and acceptability of screening through qualitative interviews with parents.
3. Explore the experiences, views and acceptability of professional stakeholders regarding screening.

## 3 METHODS

### 3.1 Design

The three aims will be addressed through four sub‐studies:

1. developing and validating a novel questionnaire for parents
2. administering validated questionnaires and the novel questionnaire to parents across Level 1 countries
3. semi‐structured interviews with parents of children who have undergone or declined screening in Level 1 countries
4. qualitative interviews with professional stakeholders in Level 1, 2 and 3 countries.

**FIGURE 2:**
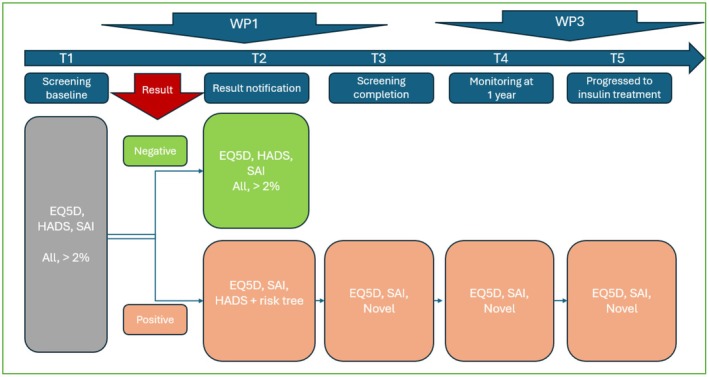
Impact questionnaires for parents of screened children from Level 1 countries: Alignment with WP1 and WP3. *Alignment with WP1*: To ensure a representative sample and mitigate potential biases, all parents/guardians participating in the WP1 screening programme will be offered the three validated questionnaires to complete. This sample will be stratified to reflect the various socio‐demographic characteristics. The questionnaires will be administered at study entry (Timepoint 1—screening baseline) and following the provision of test results (Timepoint 2—result notification). A risk decision tree will be used at T2 for ‘positive/presymptomatic’ result families to elicit parents/guardians who have high scores of anxiety and/or depression, according to the HADS questionnaire. These individuals will be promptly referred to psychological or medical professionals for the provision of psychotherapeutic support. *Alignment with WP3*: All parents/guardians participating in WP3 whose children are identified as having presymptomatic T1D will be invited to complete the three validated questionnaires, including a novel questionnaire, at Timepoints 3 (screening completion), 4 (monitoring at 1 year) and 5 (progressed to insulin treatment).

**FIGURE 3:**
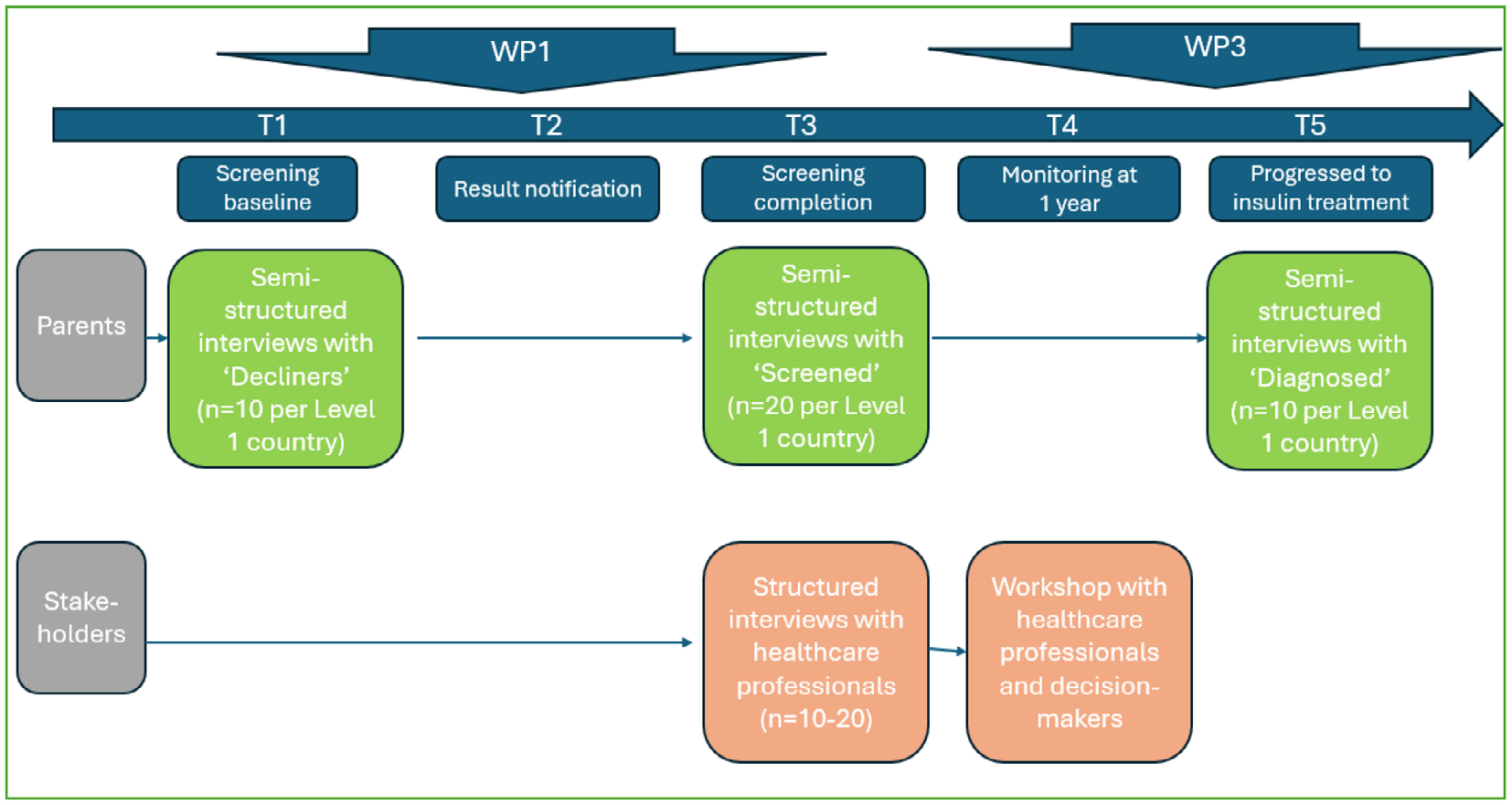
Acceptability interviews with parents and professional stakeholders: Alignment with WP1 and WP3. *Alignment with WP1*: We will conduct semi‐structured interviews with parents at three timepoints including Timepoint 1 (Screening baseline) —‘Decliners’ parents from Level 1 countries—and Timepoint 3 (Screening completion)—parents of ‘screened’ children from Level 1 countries. We will also conduct structured telephone interviews with professional stakeholders (HCPs) at Timepoint 3 (Screening completion). *Alignment with WP3*: We will carry out semi‐structured interviews with parents of ‘diagnosed’ children at Timepoint 5 (Progressed to insulin treatment). Professional stakeholders from all participating countries will be invited to the ‘final workshop’ at Timepoint 4 (Monitoring at 1 year).

Sub‐studies 1–2 are illustrated in Figure 2, and Sub‐studies 3–4 are illustrated in Figure 3.

Details of patient recruitment are provided for each of the sub‐studies below. Over and above this, all participants will be offered participation in other trials that become available through the anonymised registry (https://www.helmholtz‐munich.de/en/idf/research‐area/european‐pre‐t1d‐registry), and furthermore they will be alerted to the potential for immunotherapy medication in countries where it is available. They will also be asked if they are happy to be contacted in the future.

### 3.2 Sub‐study 1. Developing and validating a novel questionnaire for parents (English language‐based)

This sub‐study aims to develop and validate a novel patient‐reported outcome measure for parents of children participating in a general population T1D screening programme tailored to assess the psychosocial impact of presymptomatic T1D diagnosis. The novel questionnaire assesses positive/negative impact of screening. This questionnaire will be validated in English‐speaking countries (UK, US and Europe). Qualitative and validation analyses underpinning this questionnaire will be published separately.

#### 3.2.1 Participants (1)

At least 30 mothers, fathers, parents or guardians (further—‘parents’) of children who screened and tested negative or positive and those considering screening for T1D will be purposively recruited to take part in 1:1 in‐depth or focus group explorative interviews; approximately 250 parents will take part in validation of the survey and 20 parents will participate in cognitive interviews. To be eligible, an individual must meet the following criteria: (a) mother, father, parent or legal guardian of a child who has undergone or considered screening; (b) age of at least 18 years; (c) comprehends written English and (d) understands the study protocol and signs the informed consent document.

#### 3.2.2 Data collection (1)

1.1 In‐depth interviews will be conducted with eligible participants via video conferencing. The guide will explore anxiety/ distress, acceptance/understanding, behaviour and coping strategies in parent and child, including social implications and the role of the support network. This data will inform the initial development of questionnaire items.
1.2 Cognitive debriefing interviews will then be completed with eligible participants, eliciting information regarding clarity and rationale of instructions, meaning of individual items, appropriateness of response choices and any overall comments on the relevance and complexity of the questionnaire.
1.3 Survey of the novel questionnaire will be administered via Qualtrics‐enabled secure link to the participants from a wider population of families considering screening or screened to ensure representation of views.

Participating ‘Level 1’ countries' centres will translate the questionnaire into the relevant languages and ensure its cultural adaptation.

#### 3.2.3 Data analysis (1)

1.1 Focus group and individual exploratory interview data will be transcribed by a professional transcriber. NVivo will be used to pull together codes and associated quotations. Thematic content analysis^25^ will be deployed. At the completion of qualitative data analyses, all coders and investigators led by the second author (KB) will meet and generate items for the novel measure.
1.2 Cognitive debriefing data will be analysed as follows: a senior researcher (KB) will listen to all audio‐recorded interviews and summarise into key themes. A summary of the feedback will be created and discussed at length between senior researchers until consensus is reached on questionnaire revisions.
1.3 Surveys will be imported into the secure REDCap server, with unique study IDs assigned to each participant. Exploratory factor analysis (EFA) with maximum likelihood extraction, direct oblique rotation and pairwise case exclusion in SPSS version 23 will identify the factor structure of the novel measure. The number of factors will be identified using parallel analysis. Associations between demographic variables and the novel measure will be examined, using correlations, one‐way ANOVAs and independent sample *t*‐tests.

### 3.3 Sub‐study 2. Administering validated questionnaires and the novel questionnaire to parents across Level 1 countries

This sub‐study will explore the psychosocial impact of T1D screening outcome through questionnaires. Three established validated questionnaires and the novel questionnaire developed in sub‐study 1 will be used. All Level 1 countries will participate. Psychosocial impact is defined through general quality of life, anxiety and depression, and diabetes‐specific concerns and stress.

#### 3.3.1 Participants (2)

Parents of children from ‘Level 1’ countries who participated in the screening programme will be invited into this sub‐study. Inclusion criteria are mother, father, parent and guardians 18 years and older, who are willing and able to provide written informed consent. All eligible families form the general population will be invited to complete the questionnaires. We invite both parents in the family to take part in questionnaires. However, we have no control over who (in what family role) will be answering and will register responses of both parents, one parent or guardians. We expect that the main decision‐maker or family caregiver will be likely to respond. Demographic variables such as parents' age, family role, marital status, educational level, occupational status will be collected and whenever possible, we will involve underserved families (those who come from socioeconomically disadvantaged or ethnically minoritised background). It is less feasible to identify those for the questionnaires, but all possible effort will be made to involve the underserved families into acceptability interviews.

#### 3.3.2 Data collection (2)

Four validated measures will be administered either online or paper copies via participating sites in the EDENT1FI study, followed by up to two reminders 1 week apart. The willingness of parents to complete the questionnaires is a well‐known and understandable problem. We try to counter this by providing motivating information and keeping the number of questionnaires to a minimum.

Validated measures: EQ‐5D‐5L [EQ‐5D‐5L | EuroQol]^26^ assesses quality of life self‐reported as of ‘today’; HADS—Hospital Anxiety and Depression Scale—14 items^27^ identifies cases (possible and probable) of anxiety disorders and depression among patients in nonpsychiatric hospital clinics; SAI‐6—State Anxiety Inventory 6 items^28^ is a short form of 20‐item STAI adapted for assessing stress in parents of at risk of developing diabetes children; Novel questionnaire (from sub‐study 1) following validation.

It is expected that following administration of the questionnaires, a HADS‐based risk decision tree will be used to elicit parents who have high scores of anxiety and/or depression, according to the HADS questionnaire. These individuals (expected in rare cases) will be promptly referred to psychological or medical professionals for the provision of psychotherapeutic support. Refusals to respond, withdrawals and missing data will be accurately documented. Monitoring of positive result families and associated education, including parents and siblings, is part of the ‘Monitoring’ Work Package and is being published elsewhere.

#### 3.3.3 Data analysis (2)

Exploratory data analyses and graphical representations of the data will be used to check validity. The study design includes repeated measures on all outcomes; thus, linear mixed‐effect models that correspond well to this structure (e.g. Linear Mixed Models in SPSS) will be used to analyse the data. Subgroup analyses are planned by sex and age (of parents/participant); the main result will be the analyses of the pooled data and a secondary country‐specific analysis (i.e., paired and non‐paired).

Due to the intrinsic nature of the observational study (and not a clinical trial), a randomisation process is not foreseen. However, we are fully aware of the importance of constructing a sample that is representative of the general population. For this reason, we will employ statistical tools capable of addressing potential biases arising from the lack of randomisation. Specifically, multivariate models will be implemented to account for possible confounding variables, such as education level. In addition, a stratified analysis will be conducted, dividing the study population into homogeneous subgroups (strata) based on one or more confounding variables (e.g., age, education level). A dedicated missing data analysis will be conducted to address this issue. Sensitivity analyses will be performed to assess both Missing at Random (MAR) and Informative (Non‐Random) Missingness. Missing data will be evaluated at specific time points as well as longitudinally over time, in order to determine whether dropout has occurred, resulting in loss to follow‐up.

The team identified SAI‐6 as the primary measure, assessing the psychological impact at different time points. The main hypotheses are: (1) in negative families, reduced parents' stress at T2 compared with T1; (2) in positive/presymptomatic families, reduced parents' stress at T4 compared with T2. Additional hypotheses will also be tested. Where patients progress rapidly to insulin after T2, they will only have T2 and T5 responses, but we feel this will be adequate as they would have participated in education. Information on symptom recognition will be given to all participants at the time of education.

The study will be reported according to Consort guidelines.

### 3.4 Sub‐study 3. Semi‐structured interviews with parents of children who have undergone or declined screening in Level 1 countries

This sub‐study will explore the acceptability of T1D screening outcome through qualitative interviews undertaken at specific timepoints in the screening and follow‐up pathway. All Level 1 countries will participate. Acceptability will be based on qualitative self‐reports, rather than on objective measures of drop‐out/attrition rates due to the variable modes of screening and testing across the participating centres. To achieve this, we conduct qualitative semi‐structured interviews with parents. We based our approach on the combined definition of acceptability agreed upon and adopted in our previous publication (i.e., systematic review accepted by Diabetologia). It should read: acceptability in Theoretical Framework of Acceptability is understood as a multi‐faceted construct that reflects the extent to which people delivering or receiving a healthcare intervention consider it to be appropriate, based on anticipated or experiential cognitive and emotional responses to the intervention.^29^ This framework consists of seven constructs related to acceptability including affective attitude, burden, perceived effectiveness, ethicality, intervention coherence, opportunity costs and self‐efficacy. Additional to psychological interpretation of acceptability, we will also extend our approach to include physical and social/interactional experiences, as informed by COM‐B (Capability, Opportunity and Motivation = Behaviour model^30^).

#### 3.4.1 Participants (3)

Parents who have either taken part in the screening programme or declined will be recruited from Level 1 countries. They should be able to understand the consenting procedures and aims of the research. A broad range of parents will be invited for interviews, including those from minority ethnic and socioeconomically deprived groups.

We will invite two cohorts of parents: (1) those whose children took part in screening and received either positive (*n* = 10) or negative (*n* = 10) result—20 parents per country (*n* = 80); (2) those who declined their children's participation in screening for any reason,10 parents per country (*n* = 40). We estimate that these numbers will be sufficient to achieve thematic saturation. Withdrawn families are often challenging to reach. Reasons may be logistical, procedural and due to low acceptability—‘any reasons’ accounts for them all, but we will distinguish those in the process of analysis. Local centres will be recruiting decliners via community and paediatricians.

#### 3.4.2 Data collection (3)

The semi‐structured interviews will be carried out via online platforms with video recording being switched off, via telephone or face‐to‐face. The timeframe for interviews is defined as 3 months following receipt of screening result or decline. Interviews will last 40–60 min and will be driven by an interview topic guide. Parents' interviews will include one to two interviews (with either/or both mother and father) per family and their comments on the children's experiences and opinions will be elicited too. Those parents whose child tested positively and progressing to insulin‐requiring T1D will also be interviewed post‐diagnosis.

We understand that the qualitative interviews with parents of children identified as high risk may cause some distress, and parents can choose to take a break or stop the interview at any time.

#### 3.4.3 Data analysis (3)

Interviews will be audio‐recorded, transcribed, translated into English and analysed by the respective Level 1 countries. The data will be thematically analysed using the Braun and Clarke approach^31^ and informed by the combination of TFA and COM‐B theories.

### 3.5 Sub‐study 4. Qualitative interviews with professional stakeholders in Level 1, 2 and 3 countries

This sub‐study will explore the acceptability and implementation of screening with professional stakeholders through structured interviews. The interviews will be focussed on the views around barriers and facilitators and will be tailored to innovation, context and feasibility of implementation.

#### 3.5.1 Participants (4)

Healthcare professionals (HCPs), such as paediatricians, primary care practitioners and community‐based professional stakeholders, stakeholders—policy makers involved in planning and implementing the screening programme, as well as those who were not involved, will be approached. Purposive sampling will be used to select professional stakeholders to interview. The sample consists of 20 stakeholders from each country (*n* = 10 of those involved and *n* = 10 of those not involved in screening). Informed consent will be obtained from stakeholders prior to participation.

#### 3.5.2 Data collection (4)

The structured interviews (all participating countries) will be carried out via telephone or online platforms as outlined in sub‐study 2 and be informed by the Consolidated Framework for Implementation Research (CFIR).^32^ The ‘final workshop’ conducted online will aim to synthesise learning and will last 90 min. During the workshop, key professional stakeholders, researchers and decision‐makers will discuss the results of both the parents' psychological impact and acceptability of the study, share common practices, identify the country‐specific differences and deliberate on further actions. The format of the first half of the workshop will be based on SWOT (Strengths, Weaknesses, Opportunities and Threats) analysis and the second part will focus on discussion of recommendations for the implementation of future screening programs.

#### 3.5.3 Data analysis (4)

Interviews will be audio‐recorded and transcribed within each respective country as previously described for sub‐study 3. The data will be thematically analysed using a deductive–inductive approach grounded in the CFIR framework.

### 3.6 Integrative data analysis

Following independent quantitative and qualitative analyses, a mixed‐method integrative and culturally sensitive analysis will be undertaken to involve both ‘parents’ datasets. Integrative logic (mixing methods to ask questions about connecting parts, segments or layers of screening impacts) and triangulation procedures will be deployed. Among the latter, quantising qualitative data will help in representing verbal data in numerical format while qualitising quantitative data will enable the use of scores on instruments to create verbal portraits, profiles or typologies of them. Where possible, pairwise comparisons within individual cases of parents when the mean scores of the quantitative measures will be compared with the qualitatively derived ratings for the categories and themes. Dissonance, agreement or silence between independent themes originated from both methods will be explored. Finally, we will compose a matrix displaying the final list of key findings emerging from both datasets.

## 4 ETHICAL CONSIDERATIONS

The study will be conducted in accordance with local guidelines of good clinical practice that have their origin in the Declaration of Helsinki. The approval from the EDENT1FI's Ethics Advisory Board and respective countries' ethical boards/Research Ethics Committees is sought prior to the study commencement. Safeguards such as for privacy and confidentiality of data will be applied in all cases.

## 5 PATIENT AND PUBLIC INVOLVEMENT

The Patient Advisory Committee (PAC) within EDENT1FI plays a vital role in ensuring that the research remains aligned with the needs and perspectives of individuals affected by T1D and their parents. Led by BreakthroughT1D, the PAC is comprised of young individuals and parents directly impacted by T1D from eight countries, providing invaluable insights, feedback and guidance throughout all stages of the project and ensuring that the feedback is regionally and culturally informed. They actively participate in reviewing various documents, including protocols, ethical approval documents, interview guides, research proposals, and more, originating from the other WPs, WP2 and the acceptability study, in particular. The PAC convenes online every three months, or more frequently if urgent document reviews are needed, to ensure timely input and feedback as well as once per year in person at the annual EDENT1FI meetings. This regular communication and collaboration foster a deeper understanding of the patient experience, enhancing the relevance, impact and ethical integrity of the research conducted within EDENT1FI.

To ensure that patient‐facing, recruitment and education materials are child and family‐centred, the programme's PAC will review the study materials. Additionally, advisory panel members, all of whom have lived experience with T1D (affected themselves or are parents of children with T1D), will provide feedback on findings and will support dissemination efforts.

## 6 DISCUSSION

With this study, we intend to capture both quantitative and qualitative evidence to gain breadth and depth of opinions and to triangulate the extant knowledge on the psychosocial implications of T1D screening programmes. As recently suggested,^15^ attempts to separate anxiety and depression associated with screening from concurrent life stresses were limited, and the implications and acceptability of screening children need further investigation. The studies to date are of different quality with limited sample sizes. Monitoring with longitudinal aim in sight has been rarely implemented. Current population screening studies, including EDENT1FI, are ideally placed to address these gaps.

This research study is being conducted with a view to ultimately improve clinical care by providing tailored monitoring, education and offering disease‐modifying therapies. The families are not promised ‘cure’ or direct care, but they consent to the T1D screening and WP ‘Impact’ explores their experiences of it.

We also recognise that thresholds for recommending and/or mandating screening for paediatric populations based on acceptability and psychological impact rates to be identified as a result of our study are likely to be misleading and will not be pursued. We, however, provide statistical significance of 10% as derived from the impact questionnaires rather than meaningful ‘clinical’ or ‘acceptable’ significance, which we trust is inappropriate.

Sensitised to the local contexts, this mixed‐method, longitudinal study will provide evidence which will further our understanding of the wider population screening impacts in European countries. On the heels of such insight, the ‘optimal’ screening program and its acceptable variations may be distinguished and endorsed across Europe and, potentially, scale up in worldwide settings. The pathways for the best practices of clinical care with presymptomatic T1D would need to be devised following this investigation, including those based on the guidance for the closer collaboration between endocrinologists and primary care professionals,^8^ tailored education and psychological supports as well as a remediation of a wider effect on quality of life, insurance and relationships.^33^

## AUTHOR CONTRIBUTIONS

OB and PN wrote the first draft of the manuscript based on the initial proposal and team discussions. OB, KBK, JV, SG, RPD, LQ, IL, TP, JD, GP, FG, FM, VDM, KL, CM, LO, AGZ, EB, CDP and PN contributed to discussions on the protocol, reviewed and edited the manuscript. All authors approved the final version of the manuscript. PN is the guarantor of this work and, as such, takes responsibility for the integrity and accuracy of the proposed study.

## CONFLICT OF INTEREST STATEMENT

OB, KBK, JV, SG, RPD, LQ, IL, TP, JD, GP, FG, FM, VDM, KL, CM, LO, AGZ, EB, CDP and PN have no conflicts of interest to declare. JD is an employee of Sanofi. CDP is an employee of BreakthroughT1D. RD received honoraria from Sanofi (Advisory Board participation, speaker fee). PN received a consultancy fee from Sanofi. CM has the following disclosures; (1) Consulting fees: Novo Nordisk, Sanofi, Eli Lilly and Company, Novartis, Dexcom, Boehringer Ingelheim, Bayer, Roche, Abbott, Medtronic, Insulet, Biomea Fusion, SAB Bio and Vertex; (2) Honoraria: Novo Nordisk, Sanofi, Eli Lilly and Company, Dexcom, Boehringer Ingelheim, Bayer, Roche, Abbott, Medtronic and Vertex; (3) Data safety monitoring/advisory board: Novo Nordisk, Sanofi, Eli Lilly and Company, Novartis, Dexcom, Boehringer Ingelheim, Bayer, Roche, Abbott, Medtronic, Insulet, Biomea Fusion, SAB Bio and Vertex; (4) President of EASD and Vice‐President of EUD. AGZ has the following disclosures: (1) Horizon Europe, IHI Innovative Health Initiative, EDENT1FI, Grant no 101132379, (2) The Leona M. & Harry B. Helmsley Charitable Trust, EDENT1FI, Grant no. 2302–06621, (3) Sanofi France, (4) Georg Thieme Verlag KG, (5) Novo Nordisk Norway AS, (6) Deutschen Diabetes Gesellschaft e.V. (DDG), (7) Provention Bio (Rho.Inc.)Sanofi, (8) Sanofi US Services Inc., (9) ITB‐Med, (10) Sanofi‐aventis US.
